# PD140 A Strengths, Weaknesses, Opportunities, And Threats Analysis Of Prehabilitation Before Elective Surgery For Frail Elderly Patients

**DOI:** 10.1017/S0266462324003763

**Published:** 2025-01-07

**Authors:** Susanne Felgner, Tamina Fuchs, Carina Pfab, Anna Frederike Sontag, Helene Eckhardt, Wilm Quentin, Zoe Weber, Jörn Kiselev, Tanja Rombey

## Abstract

**Introduction:**

As part of the prehabilitation of elderly frail or pre-frail patients prior to elective surgery (PRAEP-GO) randomized controlled trial (NCT04418271), we are investigating the approach of multimodal prehabilitation (e.g., physiotherapy) combined with frailty screening and a shared decision-making conference for frail elderly patients prior to elective surgery. The aim of this analysis was to identify strengths, weaknesses, opportunities, and risks of the PRAEP-GO intervention to inform its implementation within the German healthcare system.

**Methods:**

This strengths, weaknesses, opportunities, and threats (SWOT) analysis was based on two expert interview studies and a realist review aiming to identify factors that might facilitate or hinder the implementation of prehabilitation from the perspectives of health service providers and patients. As part of the SWOT analysis, one author categorized these factors into external and internal factors, assessed their importance (scale of one [no/barely] to five [very high] points) and visualized it using radar plots, and assigned them to the categories of strengths, weaknesses, opportunities, or threats. Strategies were then developed to address the following combinations of the categories: strengths/opportunities, strengths/threats, weaknesses/opportunities, and weaknesses/threats.

**Results:**

In the expert interviews and the realist review, 45 facilitating and 28 hindering factors focusing on health service provision, patients, and the implementation of prehabilitation for frail patients in general were identified. In the SWOT analysis these factors were assigned to 60 internal (resource analysis) and 13 external factors (environmental analysis), and were further sorted into eight and five categories, respectively; for example, “equipment” and “staff” (these two categories were assigned the highest importance [5 points]). Strategies for utilizing strengths and opportunities included, for example, promotion of interdisciplinary cooperation between service providers. The weaknesses and risks of PRAEP-GO might be mitigated, for example, by improving infrastructures.

**Conclusions:**

A variety of factors in different categories may facilitate or hinder the implementation of PRAEP-GO. Based on preliminary results from this analysis, the identified strengths of prehabilitation outweigh its weaknesses, but when analyzing the external factors, the identified risks outweigh the benefits. Thus, the implementation of prehabilitation should be prepared by optimizing framework conditions (e.g., transportation for patients and allocation of personnel).

